# Gender and Geographic Equity in the International Association for Dental Research Awards

**DOI:** 10.1177/23800844241296829

**Published:** 2024-12-09

**Authors:** R. Lalloo, L.N. Borrell

**Affiliations:** 1 The University of Queensland, School of Dentistry, Brisbane, QLD, Australia; 2 Department of Epidemiology & Biostatistics, Graduate School of Public Health and Health Policy, The City University of New York, NY, USA; 3 Department of Surgery, Medical and Social Sciences, Faculty of Medicine and Health Sciences, University of Alcalá, Alcalá de Henares, Spain

**Keywords:** intersectionality, geography, global, awards, diversity, inclusion

## Abstract

**Objective:**

To examine the gender and geographic distribution of the International Association for Dental, Oral, and Craniofacial Research (IADR) Distinguished Scientist Awards (DSA; data: 2019–2024), group awards (data: 1982–2024), and fellowships (data: 1987–2024).

**Methods:**

Publicly available data were obtained from the IADR awards website. Information on gender and location of the awardees was gathered from photographs and affiliations if available or otherwise from online profiles or using Genderize, an online gender allocation platform.

**Results:**

Of the 99 DSA awardees in 2019 to 2024, 35% were women; 38% were from the United States, 12% were from the United Kingdom, and 11% were from Australia. Of the 35 DSA women awardees, 54% were from the United States and 11% from Finland. Of the 795 group awardees, 45% were women. Data on the location of awardees were readily available for 681 group awards; of these, 39% were from the United States and 87% were from high-income countries. Of the 113 fellowships awarded, 58% were to women. Of the 32 fellowships since 2018, 44% were for awardees from upper-middle income countries.

**Conclusion:**

Although women are overrepresented in the dental profession and the IADR has a global membership, women awardees remain underrepresented, and most awards are granted to researchers in high-income countries. These findings call attention to a deeper look at diversity, equity, and inclusion within the IADR.

**Knowledge Transfer Statement:**

Gender diversity of IADR research awards is moving in the right direction, but geographic diversity lags, with most Distinguished Scientist Awards and group awards to members in high-income countries. There is an urgent need to consider performance relative to opportunities and applications. This change in process could provide a much-needed intersectionality lens for recognizing the research performance of IADR members while increasing diversity and inclusivity.

## Introduction

The lack of gender and location diversity in the dental profession is not new (Tiwari et al. 2019). A recent study showed that among 159 editors-in-chief across 124 dental journals, 18% were women and 30% were based in the United States (Lalloo 2022). Moreover, two-thirds of editors and half of editorial board members were from the United States, the United Kingdom, Brazil, and Japan. Similarly, women are less likely to be keynote and invited speakers at annual conferences than men are. In 2018 and 2019, across dental conferences in the United Kingdom, of the 352 invited speakers, 40% were female (Heggie et al. 2021). A similar pattern has been reported in Australia (Silva and Teoh 2021) and in specific clinical disciplines, such as orthodontics (Dunn 2024). Moreover, the authorship of dental publications reported a lack of gender diversity, with only 20% of the top-cited articles being led by women between 1996 and 2015 (Haag et al. 2022). An analysis of gender and geographic diversity of the leadership across 18 global oral health organizations showed that while there has been progress toward gender diversity, most leaders were from high-income countries (Lalloo 2024). The same study also showed that 90% of global oral health conferences were held in high-income countries.

Research awards are not an exception to the observed gender gap. In 2019, an analysis of gender diversity in the Distinguished Scientist Awards (DSA) of the International Association for Dental, Oral, and Craniofacial Research (IADR) showed that between 1955 and 2018, there were 618 DSA, with only 82 (13%) awarded to women (D’Silva et al. 2019). The underrepresentation of women was observed across the 17 DSA, with women awardees ranging from 4% (Research in Periodontal Research and Research in Prosthodontics and Implants Awards) to 39% (Behavioral, Epidemiologic, and Health Services Research Award). Of the 82 women receiving awards, only one was from an upper-middle-income country, and the vast majority were from the United States (64%). Notably, D’Silva et al. provided recommendations to address and increase DSA gender diversity, such as establishing a gender quota for nominations and gender balance for the selection committee as well as ensuring the principles of diversity and inclusion for women’s representation in research programs and associations’ divisional and regional meetings. Despite these recommendations, the gender inequality pattern continues. Therefore, we aimed to extend D’Silva et al.’s analysis of DSA (2019–2024) and expand this to a similar analysis of the IADR group awards (1982–2023) and fellowships (1987–2023). Specifically, we aimed to examine gender and geographic distribution of IADR DSA, group awards, and fellowships.

## Methods

All data analyzed in this research are publicly available, either on the IADR awards website, IADR annual reports, or online profiles of awardees.

We used data from the IADR awards website for the awardees. The award name, name and surname of the winner, and the year were extracted. If photographs and affiliations were available, gender and country were captured. In addition, Genderize, an online platform software, was used to upload a database for gender assignment based on the first name of the winners. This online software assigned gender (men or women) based on the name. For names for which the platform was unable to assign a gender, an internet profile search of the awardee was conducted to assign the gender. The manually entered gender based on photographs and online profiles and Genderize assignment were matched for inconsistencies, the correct gender was allocated, and a single combined allocation was used for the analyses. In this study, only gender categories for men and women were possible.

The division and income categories of the awardees’ country were also captured. The World Bank categories for high-, upper-middle, lower-middle, and low-income countries were used to classify awardees’ locations.

DSA analysis was conducted for the recent 6 y (2019–2024), as a detailed analysis of these awards was conducted previously (D’Silva et al. 2019). A total of 99 DSA were included in the analysis. A total of 66 group awards were examined from 1982 to 2024 (at the time of submission, approximately 55% of group award winners were publicly available for 2024) and 6 fellowships from 1987 to 2024. Group travel awards were not included in this analysis. For awards not publicly listed, the IADR was contacted to request this information. The IADR provided the requested information with links and/or reports and updated the IADR website.

The gender, country, division, and income category distributions of IADR awardees were examined using frequencies and percentages. Division distribution of the membership was retrieved from annual reports for the years 2019 to 2022. For the division membership distribution, the average of the 2019 to 2023 distribution was compared with the division DSA awardees for each year (2019–2024).

## Results

Ninety-nine DSA were awarded between 2019 and 2024. Of these, 35 were awarded to women (Table 1). By year, the proportion of women awardees ranged from 24% (2022) to 41% (2020, 2021, and 2023). Of the 99 DSA awardees, 39% were awarded to the North American region, 34% to the Pan-European region, and 24% to the Asia/Pacific region. Of the 39 awarded to the North American region, 38 were from the United States. Awards were spread across 12 countries in the Pan-European region and 5 in the Asia-Pacific region. In the latter, almost half were from Australia (Table 2). Almost half of North American awardees were women, a third (35%) in Europe, and less than a fifth (17%) in the Asia-Pacific region. Of the 99 DSA awardees, 38% were from the United States, 12% from the United Kingdom, 11% from Australia, and the rest from 17 other countries (Table 2). Of the 35 women awardees, 54% were from the United States, compared with 30% for men. Almost all (31 of 35; 89%) women awardees were from the United States, the United Kingdom, Europe, and Scandinavia. This similarly reflects awardees by division, with 38% from the American, 14% from Australian-New Zealand (ANZ), 13% from Continental European, and 12% from British divisions (see Appendix Figure 1). Seventy-seven percent were awarded to members of the American, Continental European, British, and ANZ divisions, comprising half of the global membership. Compared with the membership distribution, the American, Continental European, British, ANZ, and Scandinavian divisions were overrepresented, whereas the Japanese and Chinese divisions were underrepresented (see Appendix Figure 1).

**Table 1.** Comparison of Frequencies and Percentage of Male and Female DSA Awardees, 2019 to 2024.

Year (*n*)	Women DSA Awardees, *n* (%)	Men DSA Awardees, *n* (%)
2019 (15)	5 (33.3)	10 (66.7)
2020 (17)	7 (41.2)	10 (58.8)
2021 (17)	7 (41.2)	10 (58.8)
2022 (17)	4 (23.5)	13 (76.5)
2023 (17)	7 (41.2)	10 (58.8)
2024 (16)	5 (31.3)	11 (68.7)
Overall (99)	35 (35.4)	64 (64.6)

DSA, Distinguished Scientist Award.

**Table 2.** Distribution of Distinguished Scientist Awards from 2019 to 2024, by Gender, IADR Region, and country.

IADR Region and Country	Total (%)^a^ *N* = 99	Men (%)^a^ *N* = 64	Women (%)^a^ *N* = 35	% Women Awardees (35.5%)^b^
Africa and Middle East	0	0	0	0
Asia/Pacific	24 (24.2)	20 (31.3)	4 (11.4)	16.7
Australia	11 (11.1)	8 (12.5)	3 (8.6)	27.3
China	3 (3.0)	2 (3.1)	1 (2.9)	33.3
Japan	5 (5.1)	5 (7.8)	0	0
New Zealand	3 (3.0)	3 (4.7)	0	0
Singapore	2 (2.0)	2 (3.1)	0	0
Latin American	2 (2.0)	2 (3.1)	0	0
Brazil	2 (2.0)	2 (3.1)	0	0
North American	39 (39.4)	20 (31.3)	19 (54.3)	48.7
Canada	1 (1.0)	1 (1.6)	0	0
United States	38 (38.4)	19 (29.7)	19 (54.3)	50
Pan European	34 (34.3)	22 (34.4)	12 (34.3)	35.3
Austria	1 (1.0)	1 (1.6)	0	0
Belgium	1 (1.0)	1 (1.6)	0	0
Finland	5 (5.1)	1 (1.6)	4 (11.4)	80
Germany	3 (3.0)	2 (3.1)	1 (2.9)	33.3
Ireland	1 (1.0)	0	1 (2.9)	100
Israel	1 (1.0)	1 (1.6)	0	0
Netherlands	2 (2.0)	1 (1.6)	1 (2.9)	50
Norway	1 (1.0)	1 (1.6)	0	0
Spain	1 (1.0)	1 (1.6)	0	0
Sweden	1 (1.0)	0	1 (2.9)	100
Switzerland	5 (5.1)	3 (4.7)	2 (5.7)	40
United Kingdom	12 (12.1)	10 (15.6)	2 (5.7)	16.7

a Column percentage.

b Row percentage.

Table 3 shows the 17 DSA awards by gender and income category. Of all the awardees, only 5 were not based in high-income countries, they were from 2 countries, China (*n* = 3) and Brazil (*n* = 2), and 1 was a woman. They received the H. Trendley Dean Memorial (2021), Isaac Schour (2023), Pulp Biology and Regeneration (2023), William H. Bowen (2019), and Wilmer Souder (2021) awards. There were no awards for members from lower-middle- and low-income countries. Five of the awards had equal or more women awardees than men. The IADR Gold Medal is directly related to the DSA. Since 2018, there have been 6 awarded medals, 5 to men, and all 6 from high-income countries: 3 from the United Kingdom, 2 from the United States, and 1 from Canada.

**Table 3.** Distribution of Gender and Income Category Awardees by DSA: IADR 2019 to 2024.

	Gender (*n* = 99)		Income Category (*n* = 83)	
Distinguished Scientist Award	Women, *n* = 35 (36%)	Men, *n* = 64 (64%)	High Income, *n* = 78 (94%)	Upper-Middle Income, *n* = 5 (6%)
Award in Geriatric Oral Research	1 (16.7)	5 (83.3)	6 (100)	0
Basic Research in Biological Mineralization Award	3 (50)	2 (50)	6 (100)	0
Behavioral, Epidemiologic and Health Services Award	2 (33.3)	4 (66.7)	6 (100)	0
Craniofacial Biology Research Award	3 (60)	2 (40)	5 (100)	0
Global Oral Health Research Award	1 (16.7)	5 (83.3)	6 (100)	0
H. Trendley Dean Memorial Award	3 (50)	3 (50)	5 (83.3)	1 (16.7)
Isaac Schour Memorial Award	2 (50)	2 (50)	4 (80)	1 (20)
Oral Medicine & Pathology Research Award	4 (66.7)	2 (33.3)	6 (100)	0
Pharmacology/Therapeutics/Toxicology Research Award	2 (33.3)	4 (66.7)	6 (100)	0
Pulp Biology and Regeneration Award	2 (33.3)	4 (66.7)	5 (83.3)	1 (16.7)
Research in Oral Biology Award	3 (50)	3 (50)	6 (100)	0
Research in Periodontal Disease	2 (33.3)	4 (66.7)	6 (100)	0
Research in Prosthodontics and Implants Awards	1 (16.7)	5 (83.3)	6 (100)	0
Salivary Research Award	2 (40)	3 (60)	5 (100)	0
William H. Bowen Research in Dental Caries Award	1 (16.7)	5 (83.3)	5 (88.3)	1 (16.7)
Wilmer Souder Award	1 (16.7)	5 (83.3)	5 (83.3)	1 (16.7)
Young Investigator Award	2 (33.3)	4 (66.7)	6 (100)	0

DSA, Distinguished Scientist Award; IADR, International Association for Dental, Oral, and Craniofacial Research.

Across the 66 group awards analyzed, there were 795 awardees. There were 113 fellows across the 6 fellowships (Table 4). Of the 795 group awardees, 45% (*n* = 357) were women. Of all the group awards, 22 were from the Women in Science Network (WiS). Almost all WiS awardees were from high-income countries, with the majority from the United States. Data on the locations of awardees were readily available for 681 awards (86%). Of these, 267 (39%) were from the United States, 62 (9%) from the United Kingdom, 48 (7%) from China, and 43 (6%) from Japan. The remaining awardees were distributed across 38 countries. Of the 681 awardees, 589 (87%) were from high-income countries, 86 (13%) from upper-middle-income countries, and 6 (1%) from lower-middle-income countries. Of group awardees from high-income countries, 46% were women. These percentages were 52% and 67% for upper-middle- and lower-middle-income countries, respectively. Table 5 lists the 795 group awards by the 28 scientific groups/networks. Of these, 18 had more than 9 awards, and of these, women were awarded 50% or more in 7 groups/networks. Of the 18 groups/networks, 8 had women awardees below 40%.

**Table 4.** Group Awards and Fellowships List, Years Awarded, Number of Awards, and Percentage Women Awardees: IADR.

	Years	Total Number of Awards	% Women Awardees
Group award			
AAPD Research Award	2024	1	100.0
Anthony A. Rizzo Award	1996–2012	16	6.3
Arthur Frechette Award	2010–2024	27	48.1
Aubrey Sheiham Award for Distinguished Research in Dental Public Health Sciences	2008–2024	16	37.5
Basil G. Bibby Young Investigator Award	1997–2023	26	53.8
BEHSR Outstanding Student Abstract Award	1999–2024	37	62.2
Bernard Sarnat and IADR Craniofacial Biology Award	2010–2023	13	46.2
Borrow Memorial Medal	1992–2024	33	21.2
Cariology Research Group Science Award	2016–2023	11	36.4
Cariology Research Group Student Research Award	2023	1	100.0
Colgate Oral Health Research Award	1996–2024	28	89.3
Craniofacial Biology Group Award	2013–2023	11	54.5
Crisis Prevention Institute Award for Research in Special Care Dentistry	2023	1	0.0
CTOR Award for Student Excellence in Orthodontics	2022–2023	4	100.0
CTSN Outstanding Award	2022–2024	3	100.0
DASCR Septodont Young Investigator Prize for Innovation	2011–2020	8	62.5
Digital Dentistry Research Network Student Research Award	2024	1	0.0
e-Oral Health Scientific Network Award	2024	1	0.0
Education Research Group Best Presentation Awards	2013–2019	17	64.7
Giddon Award for Distinguished Research in the Behavioral Sciences	1997–2024	30	50
GORG Post-doctoral Award	2010–2023	12	41.7
GORG Pre-doctoral Award	2010–2023	13	61.5
Growth and Development Research Award	2022–2023	2	50.0
INFORM 2020-2021 Early Career Researcher (ECR) Small Grant	2021	2	100.0
INfORM Investigator Awards	2018–2024	9	55.6
INNOVATION Award for Excellence in Orthodontics Research	2022–2023	2	100.0
Innovation in Implantology Research	2022–2023	2	0.0
Innovation in Oral Care Award	2004–2024	64	31.3
IRG Young Investigator Prize for Student Research	2010–2024	8	25.0
Ivar Mjör Award for Practice Based Research	2020–2023	4	0.0
Jonathan Ship Award	2010–2022	6	66.7
Joseph Lister Award for New Investigators	2015–2024	19	73.7
Judith Albino Award for Outstanding Research in Health Equity	2022–2024	4	75.0
Laser & Bio-photonics Group Excellence in Education Award	2024	1	0.0
Laser & Bio-photonics Group Excellence in Mentoring Award	2024	1	0.0
Laser & Bio-photonics Group Excellence in Scientific Award	2024	1	0.0
Laser & Bio-photonics Group Excellence in Young Investigator Award	2024	1	0.0
Lion Dental Research Award	2001–2024	61	36.1
MTG Outstanding Young Investigator Award for Student Research	2017–2022	30	36.7
Neuroscience Group Investigator Awards by Wiley-Blackwell Journal of Oral Rehabilitation	2011–2023	18	33.3
Nutrition Research Group Award	2010–2018	5	60.0
Orthodontic & Craniofacial Clinical and Translational Research Award	2022	1	100.0
Osteology Foundation New Investigator Award in Oral Tissue Regeneration	2020–2024	5	60.0
Pediatric Oral Health Research Group Faculty Award	2021–2024	3	66.7
Pediatric Oral Health Research Group Student Award	2018–2024	6	66.7
Pediatric Oral Health Research Group Young Investigator Award	2018–2024	6	66.7
Pediatric Oral Health Research SEA Award	2024	1	100.0
Periodontal Research Group Award - “Innovative approaches to prevention and non-surgical treatment”	2018–2024	6	16.7
Peyton-Skinner Award	1995–2024	20	20.0
Philips Oral Healthcare Young Investigator Research Grant	2007–2023	17	58.8
PRG Award in Regenerative Periodontal Medicine	2010–2023	15	13.3
Prosthodontics Research Group – The Neal Garrett Clinical Research Prize	2016–2023	7	71.4
Prosthodontics Research Group Pre-Prosthetic Regenerative Science Award for Young Investigators	2007–2023	15	33.3
Pulp Biology & Regeneration Group Awards	2009–2023	15	53.3
Pulp Biology & Regeneration Group Young Investigator Prize for Innovation	2017–2022	4	25.0
Ricardo Teles Clinical Research Award	2019–2023	5	20.0
Ryge-Mahler Award	1992–2023	20	10.0
Salivary Research Group Award	2017–2023	12	66.7
Salivary Researcher of the Year Award	1982–2021	40	30.0
Sigmund Socransky Young Investigator Award	2013–2023	11	9.1
Stephen Bayne Mid-Career Award	2017–2024	8	50.0
‘Sustainability in Oral Health’ Research Award	2023–2024	2	50.0
University of Manchester Evidence-Based Dentistry Award	2016–2022	3	33.3
Women in Science Award for Distinguished Female Mentor	2016–2024	9	100.0
Women in Science Award for Distinguished Research	2016–2023	7	100.0
Women in Science Promising Talent Award	2019–2023	6	100.0
Fellowships			
David B. Scott Fellowship	1987–2024	42	57.1
John Clarkson Fellowship	1998–2024	13	76.9
John Gray Fellowship	1993–2023	13	61.5
Norton Ross Fellowship	1992–2024	15	33.3
STAR Network Academy Fellowship	2018–2024	16	37.5
Toshio Nakao Fellowship	1995–2023	14	92.9

IADR, International Association for Dental, Oral, and Craniofacial Research.

**Table 5.** Scientific Group/Network List, Years Awarded, Number of Awards, and Percentage Women Awardees: IADR.

Scientific Group/Network	Years	Number	% Women Awardees
Behavioral, Epidemiologic and Health Services Research Group	1997–2024	98	53.1
Cariology Research Groups	1997–2023	48	45.8
Clinical and Translational Science Network	2022–2024	3	100.0
Craniofacial Biology Group	2010–2023	24	50.0
Dental Anesthesiology and Special Care Research Group	2011–2023	9	55.6
Dental Materials Group	1992–2024	48	19.6
Digital Dentistry Research Network	2024	1	0.0
DSG/IRG/MIR	2004–2024	64	31.3
e-Oral Health Scientific Network	2024	1	0.0
Education Research Group	2013–2019	17	64.7
Evidence-Based Dentistry Network	2016–2022	3	33.3
Geriatric Oral Research Group	2010–2023	31	54.8
IADR (Smile Train Cleft Research Award)	2022–2023	2	0.0
Implantology Research Group	2010–2024	10	12.5
International Network for Orofacial Pain and Related Disorders Methodology	2018–2024	11	63.6
Laser & Bio-photonics Group	2024	4	0.0
Microbiology Immunology Research Group	2001–2023	9	33.3
Mineralized Tissue Group	2017–2022	30	36.7
Network for Practice-based Research	2020–2023	4	0.0
Neuroscience Group	2011–2023	18	33.3
Nutrition Research Group	2010–2018	5	60.0
Oral Health Research Group	1996–2024	41	73.2
Orthodontic Research Group	2022–2023	9	88.9
Pediatric Oral Health Research group	1992–2024	69	47.8
Periodontal Research Group	2002–2024	70	29.7
Prosthodontics Group	1996–2024	65	38.1
Pulp Biology & Regeneration Group	2009–2023	19	47.4
Salivary Research Group	1982–2024	61	36.7
Women in Science Network	2016–2024	23	100.0

IADR, International Association for Dental, Oral, and Craniofacial Research.

Of the 113 fellowships, 66 (59%) were awarded to women. In 4 of the 6 fellowships, the majority were women (Table 4). The David B. Scott Fellowship has the most fellows, with 57% being women. Of the 64 fellows prior to 2013, 35 (55%) were women, decreasing to 8 (50%, 2013–2018) and increasing to 23 (70%, 2019–2024). Of the recent (2019–2024) 32 fellowships, 13 (41%) were based in high-income countries, 14 (44%) in upper-middle-income countries, and 5 (16%) in lower-middle-income countries. One fellow was from Venezuela, which did not have an income category.

## Discussion

Our findings suggest that gender and location diversity of awards shows some progress, but work needs to be done to reach equity relative to IADR membership. Women’s membership in the IADR is close to 50% (D’Silva et al. 2019), and women DSA awardees from 2019 to 2024 ranged from a low of 24% to a high of 41%. Of the 99 DSA awardees, 35 were women, with 54% of the women awardees coming from the United States. Of all awardees, 38% were based in the United States, 12% in the United Kingdom, and 11% in Australia. Of the 795 group awardees (1982–2024), 45% were women, 39% were from the United States, and 87% were from high-income countries. Of the 113 fellowships (1987–2024), 58.4% (*n* = 66) were women. Of the 32 fellowships since 2018, 84% of fellows were from high- and upper-middle-income countries.

D’Silva’s comprehensive analyses of DSA showed that 13% were awarded to women, with 8 of the 17 awards below 10% and only 1 (Behavioral, Epidemiologic, and Health Service Research) approaching equity at 39% (D’Silva et al. 2019). In addition, between 1999 and 2018, their analyses showed that 60 (19%) out of 316 women received this award. These analyses showed that when comparing women’s membership with the proportion of women DSA awardees from to 2015 to 2018, women received 12% to 18% of awards, underrepresenting their 41% to 46% IADR membership (D’Silva et al. 2019). Our findings (2019–2024) show some progress in the percentage of women DSA awardees, increasing to 35%. However, 5 of the 17 awards remained below 20%. D’Silva examined the location of women DSA awardees and showed that of the 82 awardees, all but 1 (from Brazil, H. Trendley Dean Memorial Award 2014) were from high-income countries, with most from the United States (64%), United Kindgom (8%), and Finland (4%). Our findings continue to show a dominance by the United States for women DSA awardees (54%) as well as for high-income countries (94%). A direct comparison of our findings on group awards and fellowships is not possible because, to the best of our knowledge, this is the first gender and location analysis of these awards.

Assuming that women’s membership has been about 50% in the recent 6 y, the percentage of women DSA awardees has remained below their membership distribution despite some progress. Gender diversity of group awards has also moved in the right direction, especially in the last 10 y and for specific awards; however, a few awards continue to be dominated by men. Fellowships are mainly awarded to women and members outside high-income countries. Although women and non-high-income members are increasingly successful in group awards and fellowships, there is a need to consider and implement strategies to address the continuing mismatch between the assumed membership of women and women’s DSA awardees. While we encourage IADR award committees to consider the D’Silva recommendations, the latter address gender diversity and not many other equity, diversity, and inclusivity indicators.

The role of the IADR in empowering women researchers highlighted 5 crucial issues: Pipeline in Oral Health Research, Economic Inequality, Workplace Harassment, Gender Bias in Scholarly Productivity, and Work-Life Balance (Ioannidou et al. 2019). The life journeys of 11 women presidents of IADR highlighted not only their significant achievements but also the challenges they experienced as women (Shaddox and Letra 2019).

Of greater concern across DSA and group awards is the dominance of high-income countries, in particular the United States, given that most IADR members are based in the United States. It is a requirement to be an IADR member to be eligible for an IADR award. Therefore, it is expected that countries where most members are from are the ones receiving most of the awards, for example, the United States. To be successful in these awards, and particularly DSA, requires significant access to capital and social resources. Research resources are often lacking (missing) in lower-middle- and low-income countries. Award panels should consider performance relative to opportunities in their selection criteria. Global awards, such as DSA, are often a reflection of privilege, reflecting the advantages some researchers will enjoy due to the structure of society around class, gender, sexuality, race/ethnicity, skin color, language, and location. Acknowledging these multifaceted identities and experiences is crucial for fostering equity, diversity, and inclusivity. This study did not assess the interaction of social identities that shapes life opportunities (Muirhead et al. 2020; Fleming et al. 2023). These all work together to either provide or deny life opportunities, including pathways to conduct cutting-edge and translational oral health research. While this analysis focuses on gender and geographic location, it is critical that organizations adopt an intersectionality lens when reflecting on their equity policies and strategies. These social identities require further investigation to ensure the true diversity of awardees across and within all equity indicators. The IADR has recently adopted a Diversity, Equity, Inclusion, Accessibility, and Belonging Statement. This statement makes a commitment “to creating an engaging environment that empowers its members to intentionally institute practices and behaviors that promote diversity, equity, inclusion, accessibility, and belonging (DEIAB).” Further evidence of the lack of gender and geographic diversity in the dental profession is reflected in an analysis of the top 2% of cited dental scientists (Ioannidis 2023).

Evidence of gender inequities in access to research funding and prominent authors of highly cited publications further highlight the need for action. For instance, of the 400 most-cited dental publications (1980–2019), men were the first or last authors in 84% (Moreno et al. 2024). The proportion of women as last authors increased from 6% to 22% in this time period, while women as first authors increased from 12% to 20%. In a separate analysis, women accounted for 20% of highly cited articles and were the last author on 16% (Haag et al. 2022). An analysis of gender differences in awards funded by the National Institute of Dental and Craniofacial Research or the National Institutes of Health showed that two-thirds of applicants and awardees were men; however, the award rates were similar for men and women (Garcia et al. 2020).

This study builds on previous DSA-only analyses. To the best of our knowledge, gender and geographic diversity of group awards and fellowships have not been previously reported. Gender was allocated to all awardees but not all locations, due to the lack of publicly available data. This requires additional effort and data to include all locations. Data vary across awards; some have photographs but often only for recent awardees; some have only names with and without locations. The potential for misclassification of gender is minimal and unlikely to alter the findings. It is also limited to men and women and is not an option for nonbinary and gender nonconforming. The gender distribution of IADR membership, overall, and by scientific group/network is not publicly available, making a direct comparison of membership and awardees impossible. DSAs are awarded to mid- to late-career researchers who are well-established in the various disciplines included in the awards. However, we used all women members as the denominator, regardless of their career stage. Therefore, the use of this denominator may have underestimated the inequities reported in our study. The location of awardees captured from the IADR awards site is at the time of the award and may be different from their prior location if they have relocated (most likely from a non-high-income country). If gathered from online profiles, this may reflect the current location and differ from their location at the time of the award. Not all group awardees for 2024 were included in this analysis.

## Conclusion

This study provides an update on the diversity of women DSA awardees and expands the analysis to include location as well as IADR group awards and fellowships. While gender diversity is improving, most awardees come from high-income countries. There is an urgent need to consider an intersectionality lens in recognizing the research performance of IADR members and esteem awards more generally across various global oral health organizations. Globally, half of the population suffers from oral conditions, mainly dental caries, and this burden is skewed toward the poor (Bernabe et al. 2020). Addressing the causes, preventing these oral conditions, promoting oral health, and improving access to care require an urgent need for radical action to end the neglect of global oral health (Peres et al. 2019; Watt et al. 2019; Guarnizo-Herreno et al. 2024). Global organizations such as the IADR and the FDI World Dental Federation play a crucial role in informing this radical action.

## Author Contributions

R. Lalloo, contributed to conception, design, data acquisition, analysis, and interpretation, drafted and critically revised the manuscript; L.N. Borrell, contributed to design, data interpretation, critically revised the manuscript. All authors gave their final approval and agree to be accountable for all aspects of work.

## Supplemental Material

sj-pptx-1-jct-10.1177_23800844241296829 – Supplemental material for Gender and Geographic Equity in the International Association for Dental Research Awards Supplemental material, sj-pptx-1-jct-10.1177_23800844241296829 for Gender and Geographic Equity in the International Association for Dental Research Awards by R. Lalloo and L.N. Borrell in JDR Clinical & Translational Research
