# Supplementary material for: Gender and Geographic Equity in the International Association for Dental Research Awards
Source: JDR Clin Trans Res. 2024 Dec 9;10(3):333–42. doi: 10.1177/23800844241296829 (PMC12166144; doi:10.1177/23800844241296829)
Supplement: sj-pptx-1-jct-10.1177_23800844241296829 – Supplemental material for Gender and Geographic Equity in the International Association for Dental Research Awards [file sj-pptx-1-jct-10.1177_23800844241296829.pptx]

## Slide 1
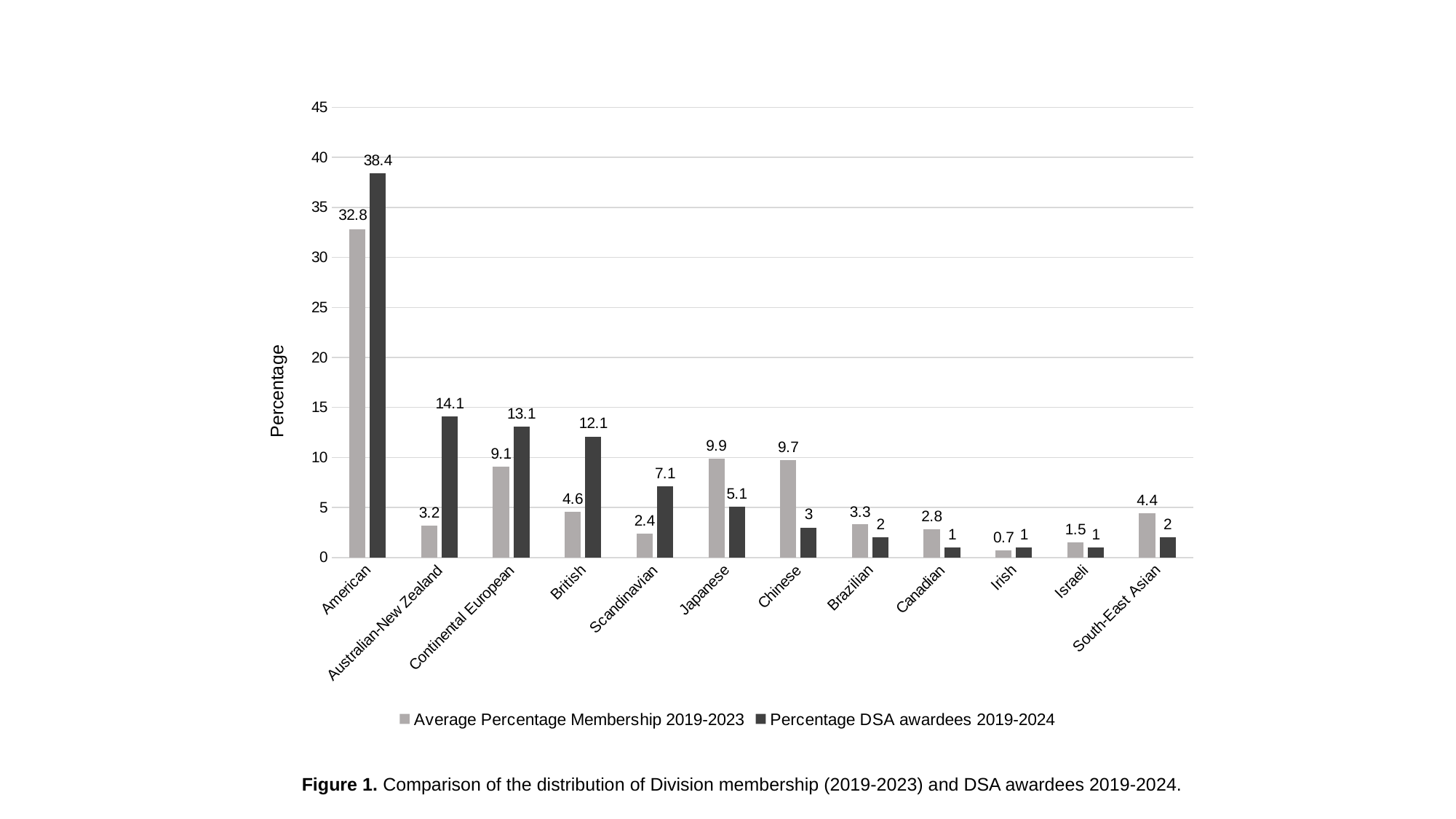

### Chart
| Category | Average Percentage Membership 2019-2023 | Percentage DSA awardees 2019-2024 |
|---|---|---|
| American | 32.8 | 38.4 |
| Australian-New Zealand | 3.2 | 14.1 |
| Continental European | 9.1 | 13.1 |
| British | 4.6 | 12.1 |
| Scandinavian | 2.4 | 7.1 |
| Japanese | 9.9 | 5.1 |
| Chinese | 9.7 | 3.0 |
| Brazilian | 3.3 | 2.0 |
| Canadian | 2.8 | 1.0 |
| Irish | 0.7 | 1.0 |
| Israeli | 1.5 | 1.0 |
| South-East Asian | 4.4 | 2.0 |Figure 1. Comparison of the distribution of Division membership (2019-2023) and DSA awardees 2019-2024.
